# Hyper-arousal vitality and its repair for attention deficit hyperactivity disorder

**DOI:** 10.3389/fpsyt.2025.1611535

**Published:** 2026-01-12

**Authors:** Deqiao Chen, Zhu Zhao, Wei Chen

**Affiliations:** 1 Department of Psychiatry, Sir Run Run Shaw Hospital, Zhejiang University School of Medicine, Hangzhou, China; 2 Center for the Study of Language and Cognition, Zhejiang University, Hangzhou, China; 3 Department of Psychology, Shaoxing University, Shaoxing, China; 4 Department of Psychology, Tongji University, Shanghai, China

**Keywords:** vitality forms, attention deficit hyperactivity disorder, ADHD, DCI–MCC circuit, social interaction, vitality form characteristic

## Abstract

Although the social challenges faced by individuals with attention deficit hyperactivity disorder (ADHD) are often attributed to attention deficits and hyperactive symptoms, their complexity necessitates re-examination from the perspective of dynamic vitality forms (VFs). VFs theory emphasizes the central role of dynamic movement styles (e.g., intensity, rhythm, spatiotemporal trajectories) in emotional transmission and intention understanding during social interactions. This study integrates neural mechanisms and behavioral characteristics to explore the abnormal manifestations of VFs in ADHD and determine their impact on social interactions. Individuals with ADHD exhibit significant dysregulation in the stability, intensity, coordination, and emotional valence of VFs, which is associated with functional abnormalities in the dorsal central insula-middle cingulate cortex (DCI–MCC) circuit that involve dopamine system dysregulation, motor coordination deficits, and emotional integration imbalances. Leveraging the dynamic plasticity of VFs, this study proposes intervention pathways such as structured motor training, impulse control, and VFs mirroring exercises to improve social functioning by utilizing the cognitive flexibility advantages of ADHD. It underscores the importance of addressing the social challenges of ADHD by moving beyond traditional behavioral frameworks and focusing on the dynamic repair of VFs.

## 1 Introduction

According to the Diagnostic and Statistical Manual of Mental Disorders (Fifth Edition) (DSM-5) (1), the diagnosis of Attention Deficit Hyperactivity Disorder (ADHD) requires meeting three core criteria: “symptoms persist for at least 6 months”, “occur in at least 2 settings (e.g., home, school, workplace)”, and “cause significant impairment in social, academic, or occupational functioning”, with symptoms onset before 12 years of age. DSM-5 explicitly classifies ADHD into three clinical subtypes: (a) Predominantly Inattentive Presentation: Requires at least 6 inattention symptoms and fewer than 5 hyperactivity/impulsivity symptoms. Clinical manifestations often include poor academic performance (e.g., procrastinating on homework, missing test questions), weak daily planning abilities, and easily neglecting others’ signals in social interactions. (b) Predominantly Hyperactive-Impulsive Presentation: Requires at least 6 hyperactivity/impulsivity symptoms and fewer than 5 inattention symptoms. Clinical features include excessive activity in early childhood (e.g., constant running, climbing dangerous objects) and frequent conflicts from intrusive behaviors in social settings (e.g., suddenly snatching others’ toys). (c) Combined Presentation: Meets at least 6 inattention symptoms and 6 hyperactivity/impulsivity symptoms simultaneously. It presents with features of both previous subtypes, such as being easily distracted and impulsively interrupting others in social interactions.

ADHD is often comorbid with other neurodevelopmental disorders, among which Autism Spectrum Disorder (ASD) and Developmental Coordination Disorder (DCD) are particularly relevant. All three are associated with immature brain development, especially involving overlapping brain regions related to attention regulation, motor control, and social cognition (e.g., prefrontal cortex, cerebellum, cingulate gyrus). The core characteristics of ASD include social communication deficits and stereotyped behaviors, accompanied by restricted, repetitive patterns of behavior, interests, or activities. The social understanding deficits of ASD can amplify the consequences of social impulsivity in ADHD. The core feature of DCD is motor coordination significantly lower than expected for peers, which impairs academic performance, daily self-care, or social functioning. The motor coordination deficits of DCD make the “hyperactivity” of ADHD present as “clumsy hyperactivity” (e.g., frequent falls while walking, unusual pen grip) rather than mere excess energy.

The clinical symptoms of individuals with ADHD often trap them in social difficulties, which stem from impaired social cognitive abilities emerging in childhood—particularly in Theory of Mind (ToM), empathy, and emotion recognition (2, 3). Current research on the social challenges of ADHD primarily focuses on cognitive and behavioral levels, such as executive function (4) and theory of mind. Barkley proposed the inhibitory control theory, which is a classic view of the executive function of ADHD (5). That is, the core functional defect of ADHD is insufficient inhibitory control and inability to effectively inhibit dominant or irrelevant behaviors. Its neural mechanism is closely related to prefrontal cortex dysfunction (6). Defects in inhibitory control directly lead to behavioral problems such as impulsive and hyperactive externalized behaviors. At the same time, it also indirectly hinders the development of social cognitive functions such as theory of mind and cognitive empathy. For example, insufficient working memory may limit the reasoning ability of children with ADHD for complex mental states (e.g., lies and sarcasm).

However, these studies yield inconsistent results (7) and fail to explain the disorder’s complexity. On the one hand, diagnostic criteria (DSM-V) oversimplify behaviors into “attention deficits” and “hyperactivity/impulsivity” neglecting context-dependent dynamic regulation. For instance, although individuals with ADHD may appear distracted in low-stimulation environments, they may exhibit exceptional focus in high-stimulation tasks (e.g., competitive games) (8). On the other hand, clinical assessment tools (e.g., Conners scales) rely on symptom frequency statistics and fail to capture temporal fluctuations. ADHD attention levels exhibit bimodal daily fluctuations (low activation in the morning, high activation at night), which static assessments cannot reflect (9, 10). The limitations of traditional explanations demand a new perspective—a dynamic, cross-temporal interactive framework.

Because social cognition is a complex process in which the target of attention shifts rapidly and is saturated with rich behavioral and contextual information, the ADHD cognitive system struggles both in selecting what to attend to and in determining the order in which information is processed. Consequently, individuals with ADHD display inappropriate behaviors, receive negative feedback from their interaction partners, and develop more enduring socio-cognitive difficulties.

The recent emergence of vitality forms (VFs) theory (11) provides a novel lens to understand human social behavior. This theory focuses on how individuals convey dynamic information—specifically, actions, including their intensity, rhythm, temporal contours, and spatial trajectories—through body movements, facial expressions, and vocalizations during social interactions. To explain VFs more clearly, we believe that VFs includes the following concepts: (i) VFs is a set of action gestalts composed of the most basic action characteristics and involving different parts of the whole body. The same VF in different situations constitutes an individual’s certain behavior characteristic, and different behavior characteristics constitute an individual’s behavior pattern/behavior style. (ii) VFs is a set of action representations that map psychological states and personality traits. (iii) VFs is a set of continuous dynamic actions. Different from unconditioned reflexes and voluntary actions, it is an action in a preconscious state. (iv) VFs is the action process between intention and result and does not necessarily affect the result. In social interaction, the vitality form is an important carrier for transmitting emotions, attitudes and intentions. When we see a person walking with brisk steps and a relaxed posture, we may perceive that he is in a pleasant or confident state; on the contrary, if a person moves slowly and stoops, it may imply that he is in a low mood or tired. It can help individuals quickly understand the inner state of others, promote emotional resonance and communication, and plays a key role in the establishment and maintenance of interpersonal relationships. The expression and perception of ADHD’s VFs may have a unique pattern and are often difficult to be captured by consciousness. Compared with language, VFs as an action gestalt can more directly convey thoughts/intentions and emotions, and is also more likely to be chosen, especially when an individual conceals or even cannot be aware of his own thoughts. For example, a warm, enthusiastic wave versus a cold, mechanical hand gesture conveys vastly different VFs and emotional messages. In social interactions, VFs serve as critical carriers of emotions, attitudes, and intentions, enabling rapid understanding of others’ mental states, fostering emotional resonance, and underpinning relationship building and maintenance. The expression and perception of VFs in ADHD may follow unique patterns, thereby influencing intersubjective interactions. This paper aims to discuss the characteristics of VFs in ADHD, their neural mechanisms, and their potential impact on social interaction processes.

## 2 Association between VFs and ADHD

### 2.1 The necessity of introducing VFs theory into ADHD

#### 2.1.1 Unresolved blind spots in previous ADHD theories

Traditional ADHD theories have mostly focused on the “what” or “why” of actions while neglecting the “how” of actions. Their core dilemmas include: (a) Vague attribution of heterogeneous behaviors: Failure to explain distinct behavioral manifestations under the same core symptoms. For instance, “hyperactivity” may present as frequent aimless small movements in some children, while others exhibit large-scale physical collisions. (b) Superficial intervention effects: Medications can reduce the incidence of inattention and impulsivity but cannot correct abnormalities in “the how of actions”—for example, a child may refrain from interrupting others but still participate in social interactions with stiff, asynchronous postures. Behavioral therapy teaches “what to do” but fails to address “how to move” leading to difficulties in generalizing skills to daily scenarios (12). (c) Difficulty explaining contradictory traits: Contrasting phenomena such as the coexistence of inattention and hyperfocus, or chaotic time perception alongside efficient performance under high pressure, are merely attributed to symptom variability without touching on the underlying differences in action regulation.(d) Lack of in-depth mechanisms for social difficulties: Social impairments are simply attributed to “poor social skills” or “deficient executive function” while ignoring ADHD-related “how” signals—for example, excessively fast movement rhythms during conversations may be perceived as “impolite” and overly weak movement intensity may be misinterpreted as “unenthusiastic”. These issues in the encoding and decoding of dynamic signals have not been covered by traditional theories.

The core breakthrough of the Vitality Forms (VFs) theory is not to negate existing mechanisms, but to theoretically fill the logical gaps in previous explanations by focusing on the perception and expression of dynamic movement characteristics, and to provide targeted intervention strategies in practice, offering a more comprehensive explanatory framework for the complex manifestations of ADHD. From the VFs theory perspective, the behavioral heterogeneity of ADHD essentially stems from abnormal combinations of VFs rather than mere symptom superposition. The study on infant vitality form play confirmed that VFs involves children’s active manipulation of dynamic features of movements/sounds, belongs to non-figurative interaction, has its own developmental trajectory (from passive perception to active manipulation), and can exist independently of other play types. Its characteristics such as movement rhythm and interpersonal synchronization have no direct correlation with ADHD’s core symptoms (e.g., inattention, impulsivity), and VFs abnormalities may persist even if core ADHD symptoms improve (13).

The core of ADHD’s social impairments lies in abnormal encoding/decoding of VFs as carriers of social signals. ADHD individuals do not lack speed in capturing social cues, but their VFs fails to match social contexts. Such encoding errors stem from a lack of the “vitality sharing” mechanism—i.e., the inability to dynamically adapt to the other party’s movement style in dyadic interactions. While medications and behavioral therapy can control inattention and impulsivity, long-standing VFs abnormalities persist as a core cause of social rejection (14). Meta-analysis also verified that traditional cognitive therapy only improves Theory of Mind (ToM) deficits but cannot correct social impairments caused by abnormal dynamic movements (7).

#### 2.1.2 The mediating role of VFs

Individuals with ADHD exhibit significant motor function abnormalities, including poor development of fine motor skills and coordinated movements, as well as higher movement variability compared to typically developing individuals. A kinematic study on children with combined-type ADHD found that patients showed higher movement error variability in limb aiming tasks, and this movement variability was significantly correlated with social communication deficits—a correlation not observed in typically developing children (15). This indicates that VFs abnormalities, centered on movement stability, force coordination, and rhythm adaptation, are independent factors directly contributing to impaired social function.

From a pathological mechanism perspective, individuals with ADHD present with abnormal development of brain regions such as the prefrontal cortex and striatum, as well as disrupted functional connectivity in the fronto-striatal neural circuit (16). These neural bases not only cause core symptoms such as inattention and impulsivity but also directly impair the integration of movement and emotion, making it difficult for patients to adjust VFs expression according to social contexts. This vicious cycle—core symptoms→neural circuit abnormalities→disrupted VFs expression→misinterpretation of social signals→social difficulties—cannot be broken by merely improving core symptoms; targeted intervention for VFs abnormalities is imperative.

Even when inattention and impulsivity are effectively controlled through medications or behavioral therapy, some patients continue to experience social rejection due to VFs-related movement characteristics (e.g., overly active limb movements, inappropriate movement rhythms) (17). They are particularly sensitive to rejection, and negative emotions triggered by social setbacks further exacerbate abnormal expression of VFs-related movement traits (18), forming an irreversible predicament. This phenomenon fully demonstrates that VFs abnormalities have become an independent mechanism maintaining social impairments, requiring specialized intervention approaches.

### 2.2 Research background

Research on VFs in neurodevelopmental disorders primarily focuses on autism. Rochat et al. were the first to experimentally demonstrate severe deficits in VFs recognition among autistic individuals, persisting from childhood to adulthood (19). Subsequent studies by Di Cesare et al. corroborated these findings (20–22). Autistic individuals also exhibit marked difficulties in VFs expression (22, 23) because, unlike neurotypical children, they cannot use specific spatiotemporal parameters to express different VFs. Because of these deficits, neurotypical individuals struggle to recognize VFs in autistic individuals, and feedback does not improve their recognition (24). Consequently, autistic individuals face profound social challenges with limited prospects for improvement.

Autism and ADHD share overlapping symptoms. Although empirical studies on VFs in ADHD are lacking, individuals with ADHD may face similar challenges in VFs recognition and expression, similar to those with autism. First, both groups exhibit deficits in social cognitive tasks (3); individuals with ADHD exhibit poor performance in theory of mind, emotional recognition, pragmatic language, and empathy (7). Second, ADHD-related motor coordination and behavioral patterns—such as redundant movements, incoherence, and poor situational adaptability (25, 26)—contribute to social difficulties. These social cognitive and behavioral traits suggest that individuals with ADHD may struggle to recognize and express VFs effectively.

### 2.3 Neural mechanisms

The dorsal central insula (DCI) is a neural hub for VFs signal processing. Recent functional magnetic resonance imaging studies (27) reveal that during action observation, the posterior superior temporal sulcus generates two information streams: one flows to the inferior parietal lobule for action goals and the other to the DCI for VFs. During action execution, the premotor cortex similarly produces two streams: one to the inferior parietal lobule for action goals and another to the DCI for VFs. Thus, whether observing or imagining/executing “gentle” or “forceful” actions, the DCI encodes VFs across visual, auditory, and motor imagery modalities, connecting with other motor cortices. The DCI is tightly coupled with the fronto-parietal mirror-neuron system and exhibits classic mirror mechanisms when processing VFs (28), thereby extending the classical mirror-neuron framework to the processing of vitality forms. By simulating others’ VFs, individuals achieve emotional resonance (e.g., perceiving anger or tenderness) (29). The DCI also integrates internal bodily states (e.g., heartbeat, respiration) with external action perceptions to form “interoceptive awareness” of VFs (e.g., intensity, rhythm) (30).

Di Cesare further highlights abnormal activation in the middle cingulate cortex (MCC) during action observation and execution (31). MCC activation supports the DCI in VFs encoding, particularly in coordinating sequential and non-sequential actions (e.g., abrupt movements). Additionally, the MCC interacts with emotional networks, enabling appropriate affective responses to others’ VFs. This indicates that the DCI–MCC circuit serves as the neural foundation for VFs perception and execution (32). Within this circuit, the DCI serves as an “emotional style processor”, translating internal sensations into dynamic action features, and the MCC functions as a “temporal coordinator”, ensuring that VFs dynamically align with environmental demands and goals.

**Figure 1:**
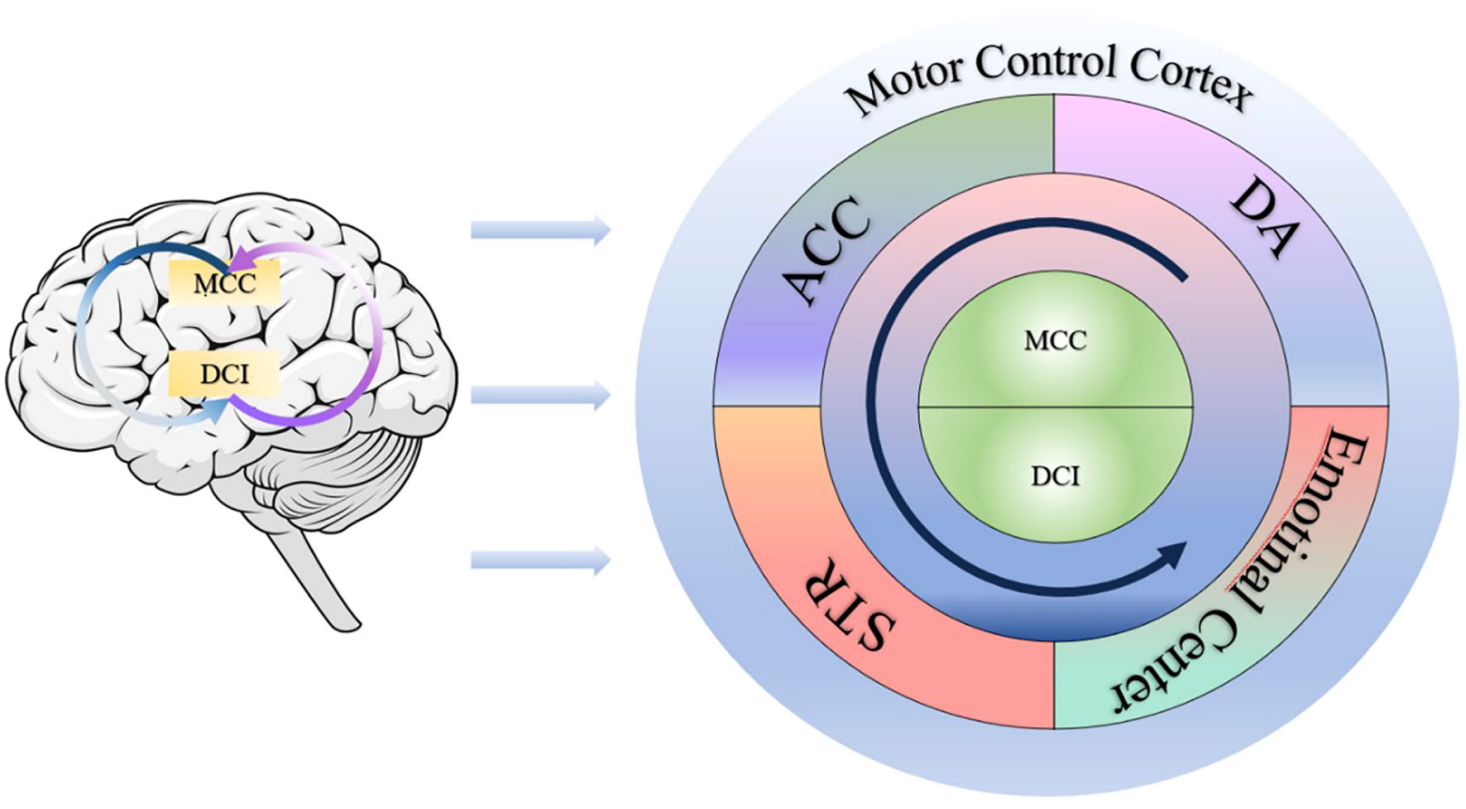
The dorsal central insula–middle cingulate cortex (DCI–MCC) circuitDCI: dorsal central insula; MCC: middle cingulate cortex; ACC: anterior cingulate cortex; DA: dopamine; STR: striatum.

Abnormal VFs in ADHD correlate with DCI–MCC circuit dysfunction, involving multiple brain structures (see **Figure 1**). Weakened functional connectivity between the insula and anterior cingulate cortex may trigger impulsive behaviors (30). The striatum, involved in social cognition and executive function (3), exhibits imbalanced reward sensitivity due to aberrant insula–striatum coupling, manifesting as heightened and reduced striatum activation in immediate and delayed reward tasks, respectively (33). This leads to initiation difficulties and impulsivity. Castellanos and Proal emphasize that default mode network–cingulate dysregulation causes attentional lapses, appearing as an “attention drift” during tasks and fragmented action sequences (34). Furthermore, dopamine system abnormalities (35) disrupt DCI–MCC circuit function, impairing motor coordination (36) and altering emotional perception (37) potentially linked to emotional centers. Ultimately, DCI–MCC signals influence prefrontal motor control cortices, contributing to VFs abnormalities.

## 3 Characteristics of VFs in ADHD

Before discussing ADHD VFs, their dimensional features need to be clarified.

Emotions and VFs are not the same. On the one hand, an emotion is typically a short-lived mental episode triggered by a specific object, whereas a vitality form is a continuous cognitive dimension present in every kind of stimulus (38). On the other hand, emotions are usually accompanied by facial expressions, whereas vitality forms are not confined to the face alone; they are revealed through the very performance or unfolding of any action. For example, “anger” is an emotion, yet it can be enacted with different vitality forms to yield finer-grained, individualized expressions. Nevertheless, the two share common ground: both carry valence—emotions can be positive or negative, and vitality forms can likewise be categorized as positive or negative. Moreover, both can coexist within the same stimulus: dance or music, for instance, is rich in vitality forms while also saturated with emotional content (39). Vitality forms and emotions are thus key non-semantic channels of human expression. Stern identifies four action dimensions—time, force, space, and directionality—with intensity and timing as core VFs dimensions (11). Chen et al. propose that VFs encompass kinematic features as well as age- and skill-related traits (14), which are refined through individuals’ life experiences. To emphasize the emotional qualities of VFs, this study categorizes their core characteristics into *action features* (stability, intensity, coordination) and *emotional features* (valence bias, stability, intensity).

### 3.1 Stability

In this context, stability refers to the coherence, predictability, and consistency of emotions and behaviors during social interactions. VFs directly convey emotions and intentions without complex inference. Stability deficits in ADHD VFs manifest as “fragmented” and incoherent expressions. However, individuals with ADHD may also exhibit greater cognitive flexibility (7), akin to autism’s empathy deficits paired with superior systematizing abilities (40, 41). In tasks requiring rapid cognitive shifts, individuals with ADHD adjust strategies more swiftly, benefiting from non-rigid thinking patterns. Such flexibility offers advantages in innovative problem-solving.

### 3.2 Intensity

Intensity dysregulation refers to variations in the force/energy of actions (e.g., speed, vigor), emotional intensity (calm vs. excitement), and social impact (active vs. passive). High-intensity actions convey anger or urgency (11), whereas low-intensity actions express care or hesitation. Unstable intensity control among individuals with ADHD leads to socially inappropriate responses (e.g., overly forceful gestures intended to be friendly).

### 3.3 Coordination

Coordination involves adapting actions to environmental or social cues, influenced by stability and intensity. Stable VFs provide predictability for interaction to promote coordination which includes movement synchrony, referring to the temporal–spatial alignment with others; adaptive adjustment, which is the dynamic modifications based on environmental feedback; and social coherence, which refers to transmitting intentions through VFs and maintaining the coherence of interaction. Individuals with ADHD struggle to adapt VFs to contextual demands, resulting in rigid movements and inefficient task-switching.

### 3.4 Valence bias

Emotional valence bias refers to preferential attention, memory, or judgment toward specific (positive/negative) valences. The unstable, uncoordinated actions of individuals with ADHD are often perceived as negative expressions. Simultaneously, such individuals may demonstrate exaggerated emotional responses (e.g., loud outbursts when happy) or misinterpret others’ emotions.

## 4 Impact of abnormal VFs on ADHD interactions

The social challenges faced by individuals with ADHD stem from abnormal VFs expression and perception. Dysregulated intensity, rhythm, and emotional valence create ambiguous or inconsistent social signals. For example, excessive intensity (e.g., abrupt gestures) may be misinterpreted as aggression and rapid emotional shifts may confuse others. Bidirectional communication breakdowns occur when individuals with ADHD struggle to decode others’ VFs because of DCI–MCC dysfunction.

**Figure 2:**
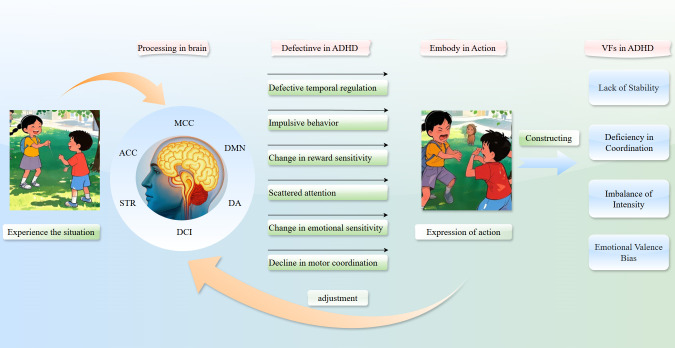
Processing and expression of attension-deficit/hyperactivity disorder (ADHD) vitality forms (VFs).

Specifically, when individuals with ADHD initiate interactions with unconventional vitality forms (e.g., high-intensity gestures to express friendliness), recipients may respond defensively because of mismatched expectations. Simultaneously, when individuals with ADHD misinterpret neutral actions as hostile, inappropriate reactions are triggered (e.g., withdrawal or impulsive retorts). Cumulative negative interactions may increase the risk of social exclusion, with peers distancing themselves because of unpredictability and individuals with ADHD adopting maladaptive strategies (e.g., suppressing natural expressions), further eroding social networks. In familial contexts, parent–child VFs mismatches (e.g., fragmented responses to calm instructions) exacerbate frustration and conflict (see **Figure 2**).

## 5 Reshaping abnormal VFs

Despite their neurobiological roots, ADHD VFs can adapt with age, experience, and targeted interventions. Their plasticity suggests the following clinical approaches in motor intervention:

### 5.1 Structured motor training

When the goal is to enhance movement stability and attention, the tasks could include: [1] Deconstructing actions into phases (e.g., “each→grasp→move”) to control timing and grip intensity (22). [2] Virtual reality focus training: Designing tasks that require sustained attention (e.g., moving objects along specified trajectories) with real-time feedback on speed and acceleration.

### 5.2 Impulse control

When the goal is to reduce impulsive VFs expressions in social contexts, the tasks could include: [1] Introducing delays (e.g., 3-second pauses) during routine activities (e.g., handing over objects). [2] Simulating social scenes through the “Stop-Signal Task”: When a specific signal (such as a whistle) is heard, immediately stop the current action (such as a raised hand posture) to train the immediate inhibitory ability of impulsive behavior.

### 5.3 Reinforcement strategy

In order to establish positive behavioral feedback, immediate rewards can be given for positive behaviors (such as stickers, extra playtime). For example, raising a hand to speak rather than interrupting in group discussions (inhibiting impulsive behavior); continuously paying attention to others’ conversations for more than 3 minutes in social activities (maintaining attention); taking a deep breath and then responding in conflict scenarios (regulating emotions).

### 5.4 Mirroring exercises

Mirror exercises involve observing and imitating others’ actions (via live demonstrations or videos) to activate mirror neuron systems. For example, parents could mirror their child’s movement speed to establish emotional resonance, then gradually guide them toward socially adaptive patterns. This leverages ADHD cognitive flexibility for gradual adaptation and conduces to promoting the development of advanced cognitive functions.

Additional methods include scenario-based simulations (e.g., role-playing) to adjust action intensity/rhythm and practice VFs adaptation across social contexts: [1] Adjusting one’s own vitality output according to the intensity of the other person’s emotions (e.g., lower the volume and slow down the speech rate when a friend is sad, and moderately raise the tone when a friend is excited). [2] Making a table of basic social etiquette (such as making eye contact when shaking hands and smiling for 3 seconds) through the “expression-action correspondence table” to avoid social misunderstandings caused by mismatched vitality (such as scaring others with excessive enthusiasm).

All of training should be recorded by video and guiding patients to observe their own dynamic performances. Training efficacy can be assessed using Conners scales or the Swanson, Nolan and Pelham Teacher and Parent Rating Scale–IV, alongside kinematic analyses (e.g., reaction speed, grip force).

## 6 Conclusion

The social challenges of ADHD extend beyond attention deficits and hyperactivity and are closely linked with dynamic abnormalities in VFs. Individuals with ADHD exhibit significant dysregulation in action stability, coordination, and emotional valence, leading to ambiguous social signaling and comprehension errors. These issues are rooted in DCI–MCC circuit dysfunction, dopamine system dysregulation, and motor control deficits. Based on the association between the core neural mechanism of ADHD and social function deficits, we emphasize promoting the development of social functions in ADHD patients through systematic behavioral interventions and reshaping their VFs. As a key neurotransmitter, the motor control, decision-making mechanism, and reinforcement learning process regulated by dopamine essentially share the brain’s reward processing circuit. The action regulation and immediate feedback mechanism in behavioral interventions precisely rebuild this neural reward pathway and transform disorderly release of vitality into an adaptive social behavior pattern. The core logic lies in: through structured behavioral training (such as action norms in social scenes and limb strategies for emotion regulation), patients activate the dopamine-mediated reward system when performing goal-oriented behaviors, gradually establishing a virtuous cycle of “behavior adjustment - positive feedback - neural reinforcement”, thereby repairing the disorderly distribution of vitality caused by delayed neural development and ultimately achieving gradual improvement in social functions.

Future research should explore interventions that harness ADHD’s cognitive flexibility to reshape VFs dynamics and offer new pathways to improve social functioning. To clarify the causal chain between VFs deficits and ADHD social impairment, we propose the following experimental designs for further verification: (i): Longitudinal follow-up study: Conduct a 2–3-year follow-up of children with ADHD, assessing core symptoms, VFs characteristics, and social function at multiple time points. Cross-lagged panel analysis will be used to test whether VFs abnormalities can predict the persistence of subsequent social impairment—rather than being driven by pre-existing core symptoms. (ii): Two-factor controlled intervention experiment: Randomly assign ADHD patients to three groups: Group A (core symptom treatment + VFs-targeted intervention), Group B (core symptom treatment only), and Group C (usual care control). By comparing the correlations between core symptom improvement, VFs function repair and social function enhancement across the three groups post-intervention, we can verify whether VFs interventions independently contribute to social improvement—an effect that cannot be achieved by core symptom treatment alone. (iii): Neuroimaging correlation study: Combine fMRI to measure changes in DCI-MCC circuit functional connectivity before and after intervention, as well as its correlations with VFs characteristics and social function.

## Data Availability

The original contributions presented in the study are included in the article/supplementary material. Further inquiries can be directed to the corresponding authors.
